# Complexity changes in functional state dynamics suggest focal connectivity reductions

**DOI:** 10.3389/fnhum.2022.958706

**Published:** 2022-09-23

**Authors:** David Sutherland Blair, Carles Soriano-Mas, Joana Cabral, Pedro Moreira, Pedro Morgado, Gustavo Deco

**Affiliations:** ^1^Facultad de Comunicación, Universitat Pompeu Fabra, Barcelona, Spain; ^2^Psychiatry and Mental Health Group, Neuroscience Program, Institut d’Investigació Biomèdica de Bellvitge, Barcelona, Spain; ^3^Network Center for Biomedical Research on Mental Health, Carlos III Health Institute, Madrid, Spain; ^4^Department of Social Psychology and Quantitative Psychology, Universitat de Barcelona, Barcelona, Spain; ^5^Life and Health Sciences Research Institute, School of Medicine, University of Minho, Braga, Portugal; ^6^ICVS/3B’s, PT Government Associate Laboratory, Braga, Portugal; ^7^Psychological Neuroscience Lab, CIPsi, School of Psychology, University of Minho, Braga, Portugal; ^8^Clinical Academic Center—Braga, Braga, Portugal; ^9^Institució Catalana de Recerca i Estudis Avançats, Barcelona, Spain; ^10^Department of Neuropsychology, Max Planck Institute for Human Cognitive and Brain Sciences, Leipzig, Germany; ^11^School of Psychological Sciences, Monash University, Clayton, VIC, Australia

**Keywords:** LEiDA, Hopf bifurcation, whole-brain model, obsessive-compulsive disorder, independent component analysis, eigendecomposition, Shannon entropy, network-based statistic

## Abstract

The past two decades have seen an explosion in the methods and directions of neuroscience research. Along with many others, complexity research has rapidly gained traction as both an independent research field and a valuable subdiscipline in computational neuroscience. In the past decade alone, several studies have suggested that psychiatric disorders affect the spatiotemporal complexity of both global and region-specific brain activity (Liu et al., 2013; Adhikari et al., 2017; Li et al., 2018). However, many of these studies have not accounted for the distributed nature of cognition in either the global or regional complexity estimates, which may lead to erroneous interpretations of both global and region-specific entropy estimates. To alleviate this concern, we propose a novel method for estimating complexity. This method relies upon projecting dynamic functional connectivity into a low-dimensional space which captures the distributed nature of brain activity. Dimension-specific entropy may be estimated within this space, which in turn allows for a rapid estimate of global signal complexity. Testing this method on a recently acquired obsessive-compulsive disorder dataset reveals substantial increases in the complexity of both global and dimension-specific activity versus healthy controls, suggesting that obsessive-compulsive patients may experience increased disorder in cognition. To probe the potential causes of this alteration, we estimate subject-level effective connectivity *via* a Hopf oscillator-based model dynamic model, the results of which suggest that obsessive-compulsive patients may experience abnormally high connectivity across a broad network in the cortex. These findings are broadly in line with results from previous studies, suggesting that this method is both robust and sensitive to group-level complexity alterations.

## Introduction

Revolution has rocked the field of neuroimaging for the past two decades. Technological development has led to previously unattainable combinations of spatial and temporal resolution, even as the discovery of organized resting state activity (Biswal et al., 1996, 1997a,1997b,^1998^; Biswal, 2012) has opened an entire new area of study. These developments have complemented one another in many lines of study, but perhaps the most notable is in the study of psychiatric disorders, where ethical concerns can make task-based or symptom provocation studies difficult (DuVal, 2004). The ability to study functional connectivity dynamics without the practical or ethical complications of symptom provocation has allowed psychiatric data collection in enormous quantity and quality. Indeed, so much data is now available that analysis has surpassed collection as the biggest challenge in neuroscience (Burns et al., 2013; Furcila et al., 2019).

Increasingly, neuroscientists have turned to mathematics and computational tools to interpret this data. The nature of neural data and the mixed backgrounds of many neuroscientists have led to the use of tools from a wide variety of mathematical fields, including statistics (Friston et al., 2006), econometrics (Friston, 2011), network analysis (Bullmore and Sporns, 2009), statistical physics (Deco et al., 2008), information theory (Pincus, 1991; Richman and Moorman, 2000), and dynamical systems (Rolls et al., 2008). The use of such tools has led to dramatic conceptual advances in the study of brain function, such as the use of network analysis to quantify structure in brain activity (Meunier et al., 2009; Shen et al., 2010) and the discovery that cognition is a distributed, rather than localized, phenomenon (Hillebrand et al., 2016; Atasoy et al., 2018). However, to paraphrase Dr. John Archibald Wheeler, as our island of knowledge grows, so too does the shoreline which surrounds it. The advances of the past two decades have revealed as many questions as answers.

One longstanding question in neuroscience and neuropsychiatry is how to quantify the complexity of the brain’s functional dynamics. While microarray studies of functional complexity are not new (Paninski, 2003; Pereda et al., 2005; Quian Quiroga and Panzeri, 2009), the whole-brain level presents two serious problems. First, even coarse neuroimaging parcellations have more than *N* = 60 regions of interest (ROIs) (Hagmann et al., 2008), and connectivity matrices have at least $\frac{N\,\left(N-1\right)}{2}$ elements (assuming symmetry and neglecting the main diagonal). The curse of dimensionality makes meaningful results difficult to find in such a high-dimensional space. Second, these regions are not generally statistically independent in time. Indeed, functional connectivity analysis relies on such interregional dependence. While these dependencies have revealed much about brain function, they also invalidate the most natural measure of functional complexity—namely the Shannon joint entropy (Shannon, 1949)—as its calculation requires statistical independence of the constituent signals. Several authors have attempted to compare the functional complexity of groups and subjects by other means (Ostwald and Bagshaw, 2011; Liu et al., 2013; McIntosh et al., 2014; Grieder et al., 2018; Zheng et al., 2020; Xin et al., 2021), but they may overlook interregional statistical dependencies and thus risk erroneous estimates of region-specific complexity. A rigorous means of quantifying the functional complexity alterations which characterize psychiatric disorders remains elusive.

In this paper, we propose a novel analysis pipeline aimed at solving these problems. We begin by adapting the Leading Eigenvector Dynamics Analysis (LEiDA) framework (Cabral et al., 2017; Figueroa et al., 2019; Lord et al., 2019) to identify a low-dimensional space which captures the temporal dynamics and complexity of functional connectivity. This requires two important innovations to the LEiDA framework. First, we develop a data-based method to estimate the state space dimensionality *a priori*. Previous studies have treated the number of dimensions as a free parameter and relied on *post facto* comparisons to determine an appropriate threshold (Cabral et al., 2017; Gu et al., 2018; Shappell et al., 2019; Vergara et al., 2020). While these methods have proven effective, they require leaving the number of groups as a free parameter. This requires multiple runs of a clustering algorithm to determine which setting is most effective. Such runs are computationally expensive, adding both time and cost to the analysis. Further, such trial-and-error approaches offer no guarantee of selecting the true number of meaningful groups. Thus, this development may improve both the precision and the efficiency of future analyses.

The use of independent component analysis represents the second major innovation to the LEiDA pipeline. Previous LEiDA analyses have used a *k*-means clustering algorithm to isolate connectivity state centroids and assign state labels to each time point. While this allows a characterization of state transition mechanics (Cabral et al., 2017; Lord et al., 2019; Vohryzek et al., 2020), *k*-means clustering suffers from two serious shortcomings which render it unsuitable for our purposes. First, *k*-means clusters generally display temporal dependencies, which make the calculation of statistical complexity extremely complex. Independent component analysis’ minimization of such dependencies (Calhoun et al., 2013) drastically simplifies these calculations. Second, most (although not all) *k*-means clustering algorithms assign only a single state to each time point. This enforces a binary, on-off image of state activity which discards much of the signal’s complexity—entirely incompatible with an algorithm designed to measure that complexity.

To alleviate these concerns, we replace the *k*-means clustering algorithm of LEiDA with independent component analysis (ICA) (Hyvärinen and Oja, 2000). ICA has been shown to maximize the temporal independence of its components (Calhoun et al., 2013) and so avoids dependencies between components. In addition, ICA does not assign a single active state to each time point, but instead estimates the activity of each state across the entire dataset. This provides a far more detailed view of how functional activity evolves in the space which these independent components define. A similar method has been proven highly effective in the context of neural spike trains (Lopes-dos-Santos et al., 2013).

These two innovations produce a space with the minimum number of independent dimensions necessary to capture meaningful patterns. Such a space makes calculating and comparing temporal complexity (as measured by the Shannon entropy) of each subject simple. Given the critical nature of ICA, we have named our analysis pipeline LEICA (Leading Eigenvector Independent Component Analysis) to differentiate it from the LEiDA framework on which it is based.

We elected to test this pipeline on a dataset (Moreira et al., 2017) consisting of obsessive-compulsive disorder (OCD) patients and number of age-, gender-, and education-matched controls (N_OCD_ = 40, N_control_ = 39). The wide prevalence and severe effects of OCD factored into this choice of dataset; with some 2.1% of the population affected each year (DuPont et al., 1995), it is a widespread, yet poorly understood disorder that causes its victims great distress. Obsessive thoughts and compulsive behaviors often hinder victims’ ability to concentrate, with predictable effects on learning and productivity (Piacentini et al., 2003; Weidle et al., 2014). These factors contribute to a high societal cost of illness (DuPont et al., 1995; Lenhard et al., 2021) and reduced quality of life for patients. Despite its prevalence, the disorder’s functional dynamics remain poorly understood; in particular, we have been unable to find any attempts to examine the functional complexity of OCD patients. In this study, we demonstrate that the obsessive-compulsive group displays elevated joint entropies compared to healthy controls. Indeed, not only can we identify which group has higher joint entropy, but also along which dimension the entropy changes.

Finally, in order to inform hypotheses on possible causes of this altered complexity, we implemented a coupled Hopf oscillator network model (Kuznetsov, 1998; Freyer et al., 2011, 2012; Deco et al., 2017b). The model estimates subject-level connectivity by fitting observed entropies. Notably, this requires the model to be trained in component space rather than the parcellation space, as the joint entropy can only be reliably calculated in this low-dimensional space. The trained model suggests that patients express enhanced connectivity in a brain-wide network, while having reduced connectivity in several small networks. It must be emphasized that link-level model results should be considered a hypothesis rather than a conclusion, as the high dimensionality of the model space makes drawing such small-scale conclusions premature. Nonetheless, the finding of general cortical hyperconnectivity coupled with targeted hypoconnectivity is consistent, and link-level results provide targets for future research. Overall, model results suggest that the LEICA method can extract alterations invisible in other spaces.

## Materials and methods

### Participants

This paper uses a dataset from a previous study at the Universidad do Minho, Portugal (Moreira et al., 2017). A detailed description may be found in that paper, but a summary is included here for completeness.

Eighty right-handed subjects (40 patients with OCD, 40 controls) participated in this study. Recruitment ensured that controls matched patients in age, sex, education, and ethnic origin. Both patients and controls were screened to remove subjects with comorbid mental health, neurological or major medical disorders (except nicotine use or dependence). Patients were all confirmed to have been using stable doses of medication for three months prior to the study. Specifically, 72.2% used selective serotonin reuptake inhibitors (SSRIs), 11.1% tricyclic antidepressants (TCA), and 16.7% a combination of these medications.

Image acquisition occurred in a 1.5 T Siemens Magnetom Avanto MRI scanner (Siemens, Erlangen, Germany) with a standard 12-channel receiver coil. Images were visually examined for artifacts and the functional data preprocessed using FSL. Slice-timing correction used the first slice as a reference, a rigid-body spatial transformation aligned the volumes of each subject with the mean volume, and motion scrubbing identified time points contaminated by significant motion. Participants with more than 20 such time points were removed from analysis. Images were then non-linearly normalized to MNI standard space and linear regression used to remove motion-related variance and signals from white matter and cerebrospinal fluid. Acquisitions were filtered with a Gaussian spatial smoothing kernel (8 mm FWHM) and a temporal band-pass filter (0.01–0.08 Hz). This frequency band has demonstrated greater reliability and functional relevance in fMRI compared to others (Biswal et al., 1995; Achard et al., 2006; Buckner et al., 2009; Glerean et al., 2012). This low frequency band has the additional advantage of averaging out physiological noise and hemodynamic response functions, as these signals have frequencies above 0.08 Hz and thus fall outside the passband of this filter. Finally, following the preprocessing, Moreira et al. (2017) extracted the mean BOLD time series of the 116 cortical, subcortical, and cerebellar regions of the Anatomical Automatic Labeling atlas (Tzourio-Mazoyer et al., 2002). As our study focuses on cortical and subcortical regions, the 26 cerebellar regions of the Anatomical Automatic Labeling (AAL) atlas were removed. A complete and ordered list of regions in this study may be viewed in Table 1.

**TABLE 1:** Table displays the 90 cortical and subcortical regions of the standard 116-region AAL parcellation (Tzourio-Mazoyer et al., 2002) in symmetrical, left-first order.

R Precentral Gyrus
R Superior Frontal Gyrus, Dorsolateral
R Superior Frontal Gyrus, Orbital Part
R Middle Frontal Gyrus
R Middle Frontal Gyrus, Orbital Part
R Inferior Frontal Gyrus, Opercular Part
R Inferior Frontal Gyrus, Triangular Part
R Inferior Frontal Gyrus, Orbital Part
R Rolandic Operculum
R Supplementary Motor Area
R Olfactory Cortex
R Superior Frontal Gyrus, Medial
R Superior Frontal Gyrus, Medial Orbital
R Gyrus Rectus
R Insula
R Anterior Cingulate and Paracingulate Gyri
R Median Cingulate and Paracingulate Gyri
R Posterior Cingulate Gyrus
R Hippocampus
R Parahippocampal Gyrus
R Amygdala
R Calcarine Fissure
R Cuneus
R Lingual Gyrus
R Superior Occipital Gyrus
R Middle Occipital Gyrus
R Inferior Occipital Gyrus
R Fusiform Gyrus
R Postcentral Gyrus
R Superior Parietal Gyrus
R Inferior Parietal Gyri
R Supramarginal Gyrus
R Angular Gyrus
R Precuneus
R Paracentral Lobule
R Caudate Nucleus
R Lenticular Nucleus, Putamen
R Lenticular Nucleus, Pallidum
R Thalamus
R Heschl Gyrus
R Superior Temporal Gyrus
R Temporal Pole: Superior Temporal Gyrus
R Middle Temporal Gyrus
R Temporal Pole: Middle Temporal Gyrus
R Inferior Temporal Gyrus
L Inferior Temporal Gyrus
L Temporal Pole: Middle Temporal Gyrus
L Middle Temporal Gyrus
L Temporal Pole: Superior Temporal Gyrus
L Superior Temporal Gyrus
L Heschl Gyrus
L Thalamus
L Lenticular Nucleus, Pallidum
L Lenticular Nucleus, Putamen
L Caudate Nucleus
L Paracentral Lobule
L Precuneus
L Angular Gyrus
L Supramarginal Gyrus
L Inferior Parietal Gyri
L Superior Parietal Gyrus
L Postcentral Gyrus
L Fusiform Gyrus
L Inferior Occipital Gyrus
L Middle Occipital Gyrus
L Superior Occipital Gyrus
L Lingual Gyrus
L Cuneus
L Calcarine Fissure
L Amygdala
L Parahippocampal Gyrus
L Hippocampus
L Posterior Cingulate Gyrus
L Median Cingulate and Paracingulate Gyri
L Anterior Cingulate and Paracingulate Gyri
L Insula
L Gyrus Rectus
L Superior Frontal Gyrus, Medial Orbital
L Superior Frontal Gyrus, Medial
L Olfactory Cortex
L Supplementary Motor Area
L Rolandic Operculum
L Inferior Frontal Gyrus, Orbital Part
L Inferior Frontal Gyrus, Triangular Part
L Inferior Frontal Gyrus, Opercular Part
L Middle Frontal Gyrus, Orbital Part
L Middle Frontal Gyrus
L Superior Frontal Gyrus, Orbital Part
L Superior Frontal Gyrus, Dorsolateral
L Precentral Gyrus

Unless otherwise noted, all figures in this study sort brain regions identically to this table. Due to space constraints, figures do not generally contain all 90 regional labels.

### Functional connectivity

#### Dynamic functional connectivity

**FIGURE 1:**
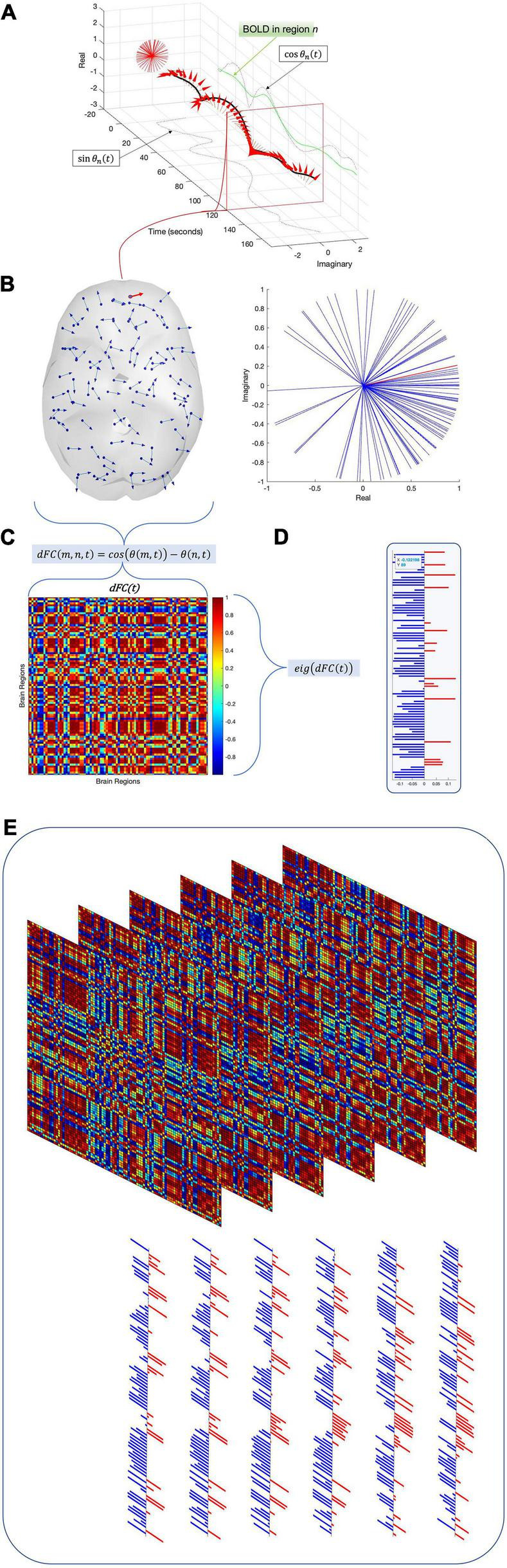
To compute time-resolved functional connectivity (dynamic functional connectivity, or dFC), each regional time series (green trace) is converted into an analytic signal using the Hilbert transform. Euler’s formula converts this analytic signal into a time-resolved phase signal **(A)** with both real and imaginary parts (dashed black traces). For each time point, the phase signals of all regions are sampled **(B)** and the cosine distance between each pair of regions is computed to produce an instantaneous functional connectivity matrix **(C)**. The leading eigenvector V_1_ of this functional connectivity matrix is then isolated **(D)**. Repeating this process for all time points and subjects across the dataset results in a 2-D array E of leading eigenvectors **(E)**. Running an eigendecomposition on E‘s autocorrelation matrix and counting the number of eigenvalues greater than the upper bound of the Marčenko–Pastur distribution reveals the number of dimensions necessary to describe the nonrandom activity in panel **(E)**.


This study uses Coherence Connectivity Dynamics (Deco et al., 2017a) to compute the dynamic functional connectivity (dFC) (Figure 1A). The remaining 90 cortical and subcortical time series were demeaned, detrended, and underwent a Hilbert transform to produce a phase time series θ, such that θ(n,t) represents the phase of region n at time t (Figure 1C). Upon computing θ, the phase coherence between regions *m* and *n* at time *t* (dFC(m,n,t)) is computed using Equation 1:

dFC⁢(m,n,t)=cos⁢(θ⁢(m,t)-θ⁢(n,t))

where cos is the cosine function. Thus, dFC(m,n,t) = 1 if the regions m and n are in phase at time t (θ(m,t)−θ(n,t) = 0,±2π), and dFC(m,n,t) = 0 if the regions are perfectly out of phase at time t (θ(m,t)−θ(n,t) = ±π). This produces a dFC array with dimensions N×N×T, where N is the number of ROIs and T represents the number of time points. Since *cos*⁡(θ) is an even function, each N×N matrix dFC(t) is symmetric.

#### Leading eigenvector analysis: Theoretical basis

The fundamental goal of the LEiDA process is to project the dominant spatial connectivity pattern dynamics into a lower-dimensional space for ease of analysis. Identifying this dominant pattern at each time point is greatly simplified by the symmetry and realness of individual dFC matrices. As symmetric and real matrices are always diagonalizable, the dFC at any time point *t* can be decomposed into


$$\text{dFC}\,\left(t\right)=V\,D\,V^{-1}$$

with *V* being the eigenvectors of dFC(*t*) and *D* the diagonal matrix of eigenvalues. As the eigenvectors of a symmetric matrix must be orthogonal, *V*^−1^=*V^T^*; thus,


$$\text{dFC}\,\left(t\right)=V\,D\,V^{T}$$

which may be equivalently written as


$$\text{dFC}\,\left(t\right)=V\,D\,V^{T}=\sum_{n}\lambda_{n}\,v_{n}\,v_{n}^{T}$$

where *v_n_* is the *n^th^* eigenvector and λ_*n*_ the *n^th^* eigenvalue of dFC(*t*). At each time point, the instantaneous FC matrix may be decomposed into a weighted sum of eigenvector outer products $v_{n}\,v_{n}^{T}$ weighted according to the respective eigenvalue λ_*n*_. Thus, finding the dominant spatial pattern at any time point simply involves finding the eigenvector with the largest eigenvalue at that time point. In addition, one may easily compute the proportion of variance which this pattern captures simply by dividing the leading eigenvalue by the sum of all eigenvalues:


$$\rho =\frac{\lambda_{l}}{\sum_{n}\lambda_{n}}$$

Previous work demonstrates that the leading eigenvector consistently represents more than 50% of data variance (Cabral et al., 2017; Lord et al., 2019), a finding confirmed in the present study. Further, experiments with the use of additional eigenvectors demonstrated no improvement in performance or clinical interpretability. The authors thus believe that a single eigenvector is sufficient to represent functional connectivity dynamics.

This compression has three distinct advantages for further signal analysis. First, by compressing each N×NdFC(t) matrix to an N×1 vector p_l_, this method reduces sample dimensionality from $\frac{\text{N}\,\left(N-1\right)}{2}$ to N . Second, the primary connectivity pattern should contain virtually no noise, as noise components generally appear in trailing eigenvectors. Finally, previous work in spectral community detection (Newman, 2006; Leicht and Newman, 2008) has demonstrated that the leading eigenvector p_l_(t) can separate brain regions into communities based on the sign of each region r ∈ p_l_(t), with the magnitude of r indicating that assignment’s “strength”. Thus, transforming the dFC(t) matrix to p_l_(t) converts interregional phase-locking values into regional community assignment values. Put another way, the leading eigenvector of an FC matrix naturally separates network nodes into two mutually opposing communities.

#### Leading eigenvector analysis: Application

We adapt the LEiDA (Cabral et al., 2017; Figueroa et al., 2019; Lord et al., 2019) by examining only the leading eigenvector **v**_l_(t) of each *N*×*N***dFC**(t) matrix. At each time point, the leading eigenvector of the *N*×*N***dFC**(t) is extracted (Figure 1D); once the leading eigenvectors of all time points have been extracted, they are concatenated horizontally to form a space-time matrix **E** (Figure 1E). Each row *r* of **E** represents one brain region r, and each column *t* contains the leading eigenvector **v**_l_(t) for time *t*. The laws of linear algebra render **v**_l_(t) and −**v_l_**(t) equivalent, so we follow the convention that most elements in each eigenvector should be negative (Figueroa et al., 2019).

#### Component detection

To find the communities that recur above chance, we must determine a significance threshold for regional co-activation. Although surrogate methods, e.g., a permutation test, can establish such a threshold, they are slow and computationally intensive. We propose a far cheaper and more elegant method based on autocorrelation matrix eigenvalues (Peyrache et al., 2009, 2010). It has been established for several decades that if an *m*×*n* matrix **M** has statistically independent rows (as would be expected for uncoupled noisy oscillators), the eigenvalues of its autocorrelation matrix follow the Marčenko–Pastur distribution (Marčenko and Pastur, 1967). Crucially, this distribution has analytically tractable limits


$$\lambda_{\text{min}}^{\text{max}}=\sigma^{2}\,{\left(1\pm \sqrt{\frac{1}{\text{q}}}\right)}^{2}$$

where σ is the standard deviation of **M** and $\text{q}\equiv \frac{\text{n}}{\text{m}}\geq 1$. Thus, if communities do not recur over time, the eigenvalues of **E**′s correlation matrix should lie within the limits imposed by $\lambda_{\text{min}}^{\text{max}}$. Conversely, should any communities of **E** recur at a rate significantly above chance, a corresponding number of eigenvalues of the correlation matrix of **E** should exceed the upper limit λ_max_. This method has been validated in the spike activity context (Lopes-dos-Santos et al., 2011, 2013) and in a previously published fMRI study (Deco et al., 2019). In the present dataset, it detects 12 components (Figure 4A).

**FIGURE 2:**
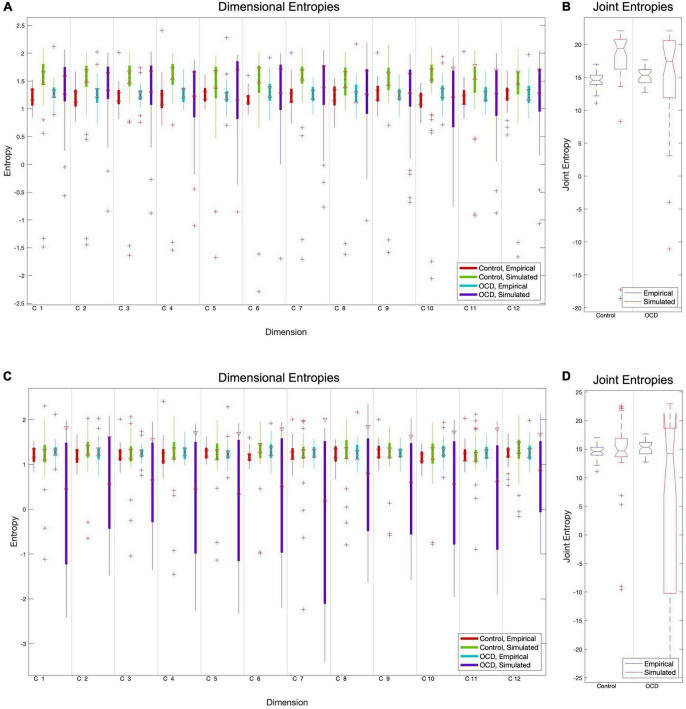
The particle swarm fitting algorithm, like most optimization algorithms, minimizes a cost function to determine how well the model predicts real data. We chose the Euclidean distance between empirical and simulated entropy vectors as a cost function due to its conceptual simplicity and confirmed its superiority versus absolute maximum distance. Comparisons of component-level entropy distributions pre-fit **(A)** and post-fit **(C)** demonstrate that this method does improve the model for controls. Comparisons of pre-fit **(B)** and post-fit **(D)** joint entropy confirm this. While optimization brings the mean entropies of patient models closer to those of empirical subjects, its performance is quite inconsistent in this group. This is reflected in the extremely high variance in post-optimization dimensional and joint entropies **(C, D)**.

**FIGURE 3:**
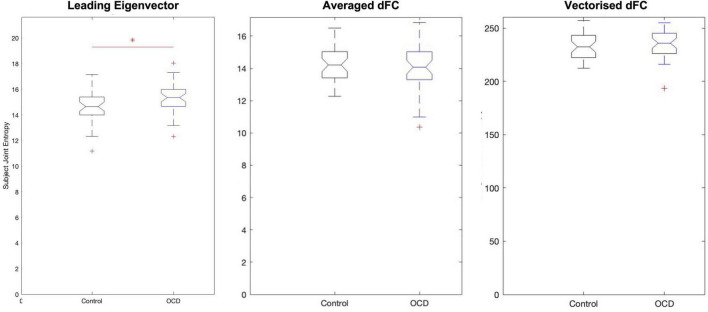
Analysis of eigenvector-based component time series (**T**_E_) shows that obsessive-compulsive patients display substantially higher joint entropy than age-, gender-, and education-matched controls. On average, controls display a joint entropy of 14.5695 ± 1.2473, while patients display a mean joint entropy of 15.2214 ± 1.1535. Neither spatial average-based components nor vectorized dFC-based components display group-level changes.

**FIGURE 4:**
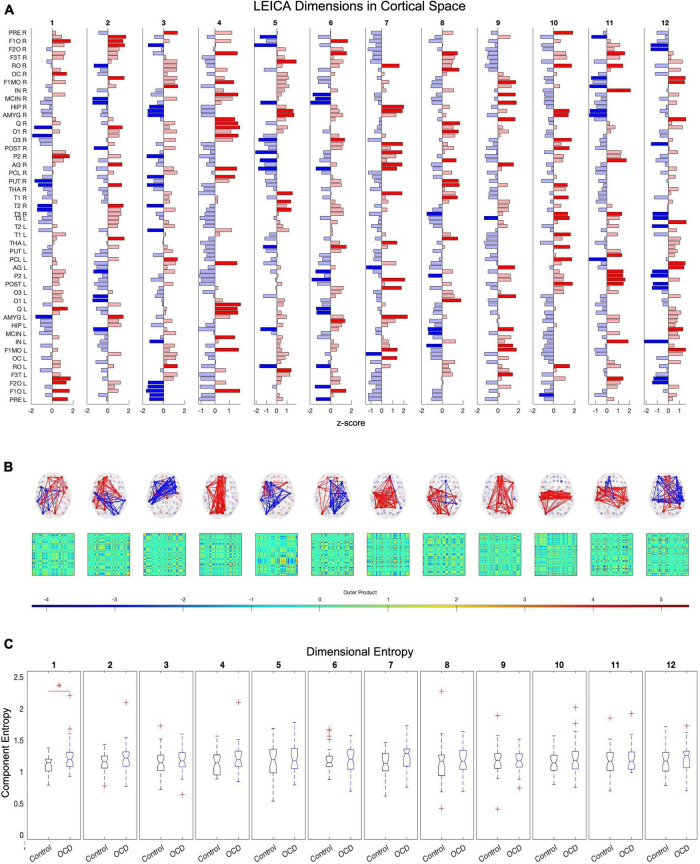
Twelve of the eigenvalues of E’s autocorrelation matrix exceed the upper limit of the Marčenko-Pastur distribution, suggesting that 12 dimensions are necessary to capture E’s activity. Independent component analysis reveals how these dimensions map to brain regions **(A)**. Map weights have been converted into z-scores for this figure and regions with a weight z < 1.3 are depicted in faded color. Plotting these mapping vectors in the brain and as connectivity **(B)** reveals that the trailing dimensions (9, 10, 11, and 12) display notable homotopic symmetry, while leading dimensions are strongly asymmetric. Finally, group-level entropy analysis shows that the first dimension displays significantly higher entropy in obsessive-compulsive patients than in controls **(C)**. Note that dimensions are ordered according to average activity level across the dataset.

#### Component extraction

Upon finding the total number of recurrent communities with the Marčenko–Pastur distribution, we utilize the fastICA algorithm (Laubach et al., 1999; Hyvärinen and Oja, 2000) to extract these communities and their activity time courses from the matrix E . Since the fastICA algorithm requires the user to manually specify the number of independent components, the Marčenko–Pastur distribution threshold is crucial to providing an objective, data-driven metric for the number of components.

After computing **E**′s covariance matrix, 12 eigenvalues were found to surpass the Marčenko–Pastur upper bound. ICA was then run to extract these 12 distinct and temporally independent components (Figure 4A). As fastICA can only extract the magnitude of an independent component, not its sign, the spatial map’s positive and negative signs should be understood to represent relative orientations rather than absolute weights.

#### Entropy analysis

Independent component analysis was selected as a clustering algorithm because, by definition, it minimizes the statistical dependencies between components. This should completely—or at least almost completely—prevent the temporal dependencies between components. If this is the case, then the joint entropy over all components is simply the sum of the individual components’ Shannon entropies (Cover and Thomas, 2005):


$$\text{H}\,\left({\text{C}}_{1},\ldots ,{\text{C}}_{\text{N}}\right)=\sum_{\text{j}=1}^{\text{N}}\text{H}\,\left({\text{C}}_{\text{j}}\right)$$

It is possible to compute the joint entropy of each subject by computing the Shannon entropy of each component’s activation time series and summing them. This allows the construction of a distribution of subject joint entropies, which can then be analyzed for group-level differences.

#### Group comparisons

We search for group-level differences using a difference-of-means permutation test (Krol, 2021) with 10,000 permutations, and provide multiple-comparison correction *via* the false discovery rate (Benjamini and Hochberg, 1995). The Bonferroni (1935) and Sidak (1967) thresholds verify these results.

### Effective connectivity

#### Brain network model

The brain network consists of the 90 cortical and subcortical nodes (regions) of the AAL parcellation, coupled according to the standard 90-region AAL connectivity template **C**. Internal node dynamics are modeled as the normal form of a supercritical Hopf oscillator (Deco et al., 2017b). This produces


$$\frac{{\text{dx}}_{\text{j}}}{\text{dt}}={\text{x}}_{\text{j}}\,\left(\alpha_{\text{j}}-{\text{x}}_{\text{j}}^{2}-{\text{y}}_{\text{j}}^{2}\right)-\omega_{\text{j}}\,{\text{y}}_{\text{j}}+\text{G}\,\sum_{\text{i}}{\text{C}}_{\text{ij}}\,\left({\text{x}}_{\text{i}}-{\text{x}}_{\text{j}}\right)\,\beta \,\eta_{\text{j}}\,\left(\text{t}\right)$$

$$\frac{{\text{dy}}_{\text{j}}}{\text{dt}}={\text{y}}_{\text{j}}\,\left(\alpha_{\text{j}}-{\text{x}}_{\text{j}}^{2}-{\text{y}}_{\text{j}}^{2}\right)-\omega_{\text{j}}\,{\text{x}}_{\text{j}}+\text{G}\,\sum_{\text{i}}{\text{C}}_{\text{ij}}\,\left({\text{y}}_{\text{i}}-{\text{y}}_{\text{j}}\right)\,\beta \,\eta_{\text{j}}\,\left(\text{t}\right)$$

where C_ij_ is the connection strength from j to i and G represents global coupling efficiency. ω_j_ is estimated directly from the BOLD time series by extracting the dominant frequency of node j within the band of 0.01 to 0.08 Hz. α and G are set to the initial values of α = 0 and G = 0.2, in line with previous work (Deco and Kringelbach, 2016; Deco et al., 2017b).

#### Particle swarm optimization

The connection strengths **C**_ij_ are optimized using the population swarm algorithm (Kennedy and Eberhart, 1995; Erik et al., 2010; Mezura-Montes et al., 2011). This algorithm simulates a population of individual particles moving in random directions within an N-dimensional space, where N is the number of free parameters. At each optimization step, each particle can continue exploring the space, move to its optimal prior position, or move to the global optimal prior position. The model is then tested using the new positions of each particle as parameters, and the individual and global optima are updated as necessary.

#### Cost function

The particle swarm algorithm seeks to minimize the difference between simulated and empirical data distributions. We quantify this difference as the Euclidean distance between entropy distributions:


$$d\,\left(S,E\right)\,\sqrt{\sum_{j=1}^{N}{\left(S\,\left(j\right)-E\,\left(j\right)\right)}^{2}}$$

After simulating a BOLD signal, this simulated signal is separated into components using the mixing matrix W, and the Shannon entropy of each component is computed. The Euclidean distance between the simulated entropy distribution and its empirical counterpart is used as the optimization cost function, which guides the particle swarm algorithm’s estimates for optimal model parameters. Pre-fit and post-fit cost function distributions are shown in Figure 2.

### Network analysis

Our study’s goal is to find network-level connectivity changes in obsessive compulsive disorder patients. To this end, we apply two group-level analyses to the connectivity estimates obtained in the previous section.

#### Network-based statistic

The network-based statistic (NBS) is a component detection approach (Zalesky et al., 2010) with substantially greater power than traditional family-wise error (FWE) correction. Unlike traditional FWE correction, the NBS tests the significance of an effect’s size rather than its magnitude. This drastically reduces the multiple-comparison correction and allows the estimation of an empirical null distribution *via* a permutation test.

Upon estimating the effective connectivity of each subject, we run a group-level comparison with the NBS to search for significant connectivity changes in obsessive compulsive disorder. As control parameters, we used a significance threshold of *t* = 4.5 and a standard case-comparison contrast. Additional significance thresholds of *t* = 4, 5, and 5.5 were also tested, the results of which results may be viewed in the Supplementary Figures.

#### Degree strength analysis

In addition to the NBS, we run a group-level comparison of the node strengths. Specifically, we test for differences in strength between groups for each node in the effective connectivity network. The directed nature of effective connectivity required that both in- and out-strength be examined.

### Comparison analyses

To compare LEICA’s efficacy to more familiar methods, we repeated the above analyses with two other versions of the dynamic functional connectivity array. The first such comparison simply consists of the vectorized upper triangle of each **dFC**(t), concatenated to form a space-time array $\left(\frac{N\,\left(N-1\right)}{2}\times T\right)$. The second comparison consists of the spatial average of each **dFC**(t), likewise concatenated to form a space-time array (*N*×*T*). Pre- and post-eigendecomposition steps are identical for all inputs.

## Results

### Functional analysis

#### Dynamic functional connectivity

Both control and patient time series are parcellated according to the AAL atlas (Tzourio-Mazoyer et al., 2002). Each subject’s dynamic functional connectivity is computed using Coherence Connectivity Dynamics (Deco et al., 2017a). Analysis is restricted to the cortical and subcortical regions; as such, the 26 cerebellar regions of the AAL atlas are discarded. The resultant three-dimensional array must be converted into two dimensions for further analysis. Three methods are tested. In the first method, we extract the leading eigenvector (90×1) of each time point’s connectivity matrix. The eigenvectors of all time point are then concatenated to form a subject-level 90×175 eigenvector time series **E**. In the second method, each time point’s connectivity matrix is averaged horizontally, and the resulting average coherence vectors (90×1) are concatenated to form a subject-level 90×175 mean coherence time series **M**. Finally, each time point’s connectivity matrix is vectorized to form a 4005×1 connectivity vector, and these vectors are again concatenated to form a subject-level 4005×175**dFC** time series (as each connectivity matrix is symmetric and the main diagonal neglected, only the upper triangle is vectorized).

#### Functional dimensions

To determine the number of dimensions necessary, all subjects’ time series are concatenated and the autocorrelation matrix of this global time series array calculated. The number of significant dimensions is then the number of eigenvalues in the autocorrelation matrix which exceed the upper bound of the Marčenko–Pastur distribution (Marčenko and Pastur, 1967). Applying this method to the eigenvector time series **E** identifies 12 independent dimensions across the resting state of all subjects (Figure 4A). ICA can then convert the 90-dimensional eigenvector time series **E** into its 12-dimensional representation **T**_*E*_ (Lopes-dos-Santos et al., 2011, 2013) (Figure 4B). Repeating this process for the vectorized **dFC** produces the 347-dimensional representation **T**_*F*_, and the spatially averaged **M** produces the 11-dimensional representation **T**_*M*_.

#### Joint entropy

Since each time series in the low-dimensional space is statistically independent, each dimension’s Shannon entropy may be calculated (Singh et al., 2003; Delattre and Fournier, 2017) independent of the others’. Computing the subject-level Shannon entropy of each substate’s time series results in a *D*×*S* array of entropy values for patients and controls, where *S* is the number of dimensions and *S* the number of subjects per group. This format means that computing the subject-level joint entropy simply requires summing along this array’s first dimension. This produces two 1×*S* joint entropy distributions, which can be compared with any standard statistical test. Applying this process to eigenvector-based entropy scores again shows elevated entropy in patients relative to controls (*p* = 0.0119, Hodges’ *G* = –0.5833) (Figure 3). However, the joint entropy distributions of **T**_*F*_ and of **T**_*M*_ display no significant group-level differences. Eigenvector-based analysis thus appears to preserve the information of the full signal while reducing dimensionality almost 30-fold—–a crucial consideration, as the curse of dimensionality states that patterns become exponentially harder to detect as dimensionality increases.

#### Dimension-specific entropy

To determine whether alterations in entropy concentrate in specific dimensions, we started with the same *D*×*S* patient and control arrays of entropy values as the previous section. Each row of these arrays was compared and corrected with the false discovery rate. As above, this analysis was run for all three compression methods: **T**_E_ (eigenvectors), **T**_F_ (uuncompressed), and **T**_M_ (spatial average). Only the eigenvector-based representation (**T**_E_) detects a significant alteration along any dimension, specifically the first (ordered according to mean activity). This dimension consists of paired anticorrelated communities and both display significantly higher entropy in patients than in age-, gender-, and education-matched controls (Figure 4C).

In component space, we find that one LEICA component displays higher entropy in patients than in controls (1.1818 ± 0.1401, 1.3075 ± 0.2276, *p* = 0.0020, Hedges’ *g*−0.6634). This substate consists of two opposing communities, with the sign of each brain region denoting to which community that region belongs and the magnitude of that region’s weight denoting the strength of its association with that community (see Table 2 for a list of implicated regions). We opted to concentrate on regions with absolute *z*-scores above 1.3 (|**z**| > 1.3) (Figure 4A). Under these constraints, the first community contains the left precentral gyrus, left and right frontal superior cortex (orbital), left middle frontal gyrus (orbital), the left inferior frontal gyrus (opercular), left cuneus, right olfactory bulb, and right inferior parietal gyrus. Its opposite number includes the right lingual gyrus, right occipital medial gyrus, right putamen, right pallidum, left amygdala, right middle temporal gyrus, and right temporal pole of the middle temporal gyrus (Figure 4). This result survives both the false discovery rate and the Sidak multiple comparison correction.

**TABLE 2:** Table displays the regions of the first dimension with absolute z-scores exceeding 1.3 (|z| > 1.3).

Component 1 (*z* > 1.3)	
**Positive**	**Negative**
L Precentral Gyrus	L Amygdala
L Superior Frontal Gyrus, Orbital Part	R Temporal Pole: Middle Temporal Gyrus
L Middle Frontal Gyrus, Orbital Part	R Middle Temporal Gyrus
L Inferior Frontal Gyrus, Opercular Part	R Lenticular Nucleus, Pallidum
L Cuneus	R Lenticular Nucleus, Putamen
R Inferior Parietal Gyri	R Middle Occipital Gyrus
R Olfactory Cortex	R Lingual Gyrus
R Superior Frontal Gyrus, Orbital Part	

The sign of each regional weight indicates to which of two communities it belongs, with the magnitude of its weight indicating its centrality to that community. Regions with absolute z-scores exceeding 1.3 (|z| > 1.3) can be considered core nodes in a more distributed network which covers the entirety of the brain space.

### Connectivity model

#### Network-based statistic

In order to hypothesize on causes for these shifts in dynamical richness, we fit a networked Hopf model (Deco et al., 2017b) to each subject’s entropy profile. After obtaining subject-level effective connectivity profiles from these models, we applied the network-based statistic (NBS) (Zalesky et al., 2010) to determine which, if any, connections display significant group-level alterations. In addition, we examined the in-strength and out-strength of each node for significant alterations between groups. Only the eigenvector-based decomposition produced a generative model which displays significant group-level alterations in network connectivity; the spatially averaged and uncompressed decompositions failed to find meaningful results.

**FIGURE 5:**
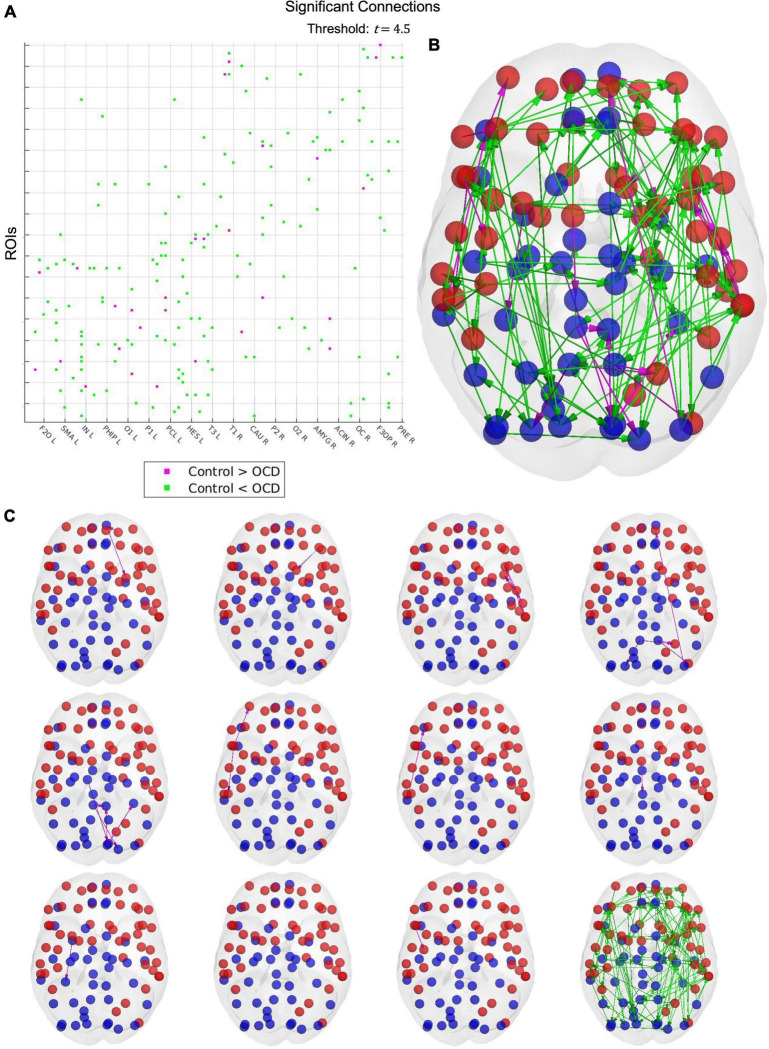
Results from the network-based statistic. A t-statistic threshold of 4.5 returns 12 connected components **(C)**, visualized together as a connectivity matrix **(A)** and in cortical space **(B)**. Cyan links indicate that the connection is stronger in OCD patients than in healthy controls, while magenta links indicate the converse. Although only one connected component displays increased strength in patients, this component includes 87 of the 90 cortical nodes in the AAL parcellation, suggesting that obsessive-compulsive disorder may be characterized by widespread cortical hyperconnectivity. The 11 control-biased components, by contrast, consist of between one to six links, with larger components tending to concentrate in small topographical areas. Notably, many regions displaying depressed connectivity in patients are known to be involved in top-down control and impulse inhibition. OCD may thus be characterized by localized disruptions in top-down inhibitory activity, which may explain the widespread hyperconnectivity observed in patients.

Results from the network-based statistic depend upon the *t*-statistic chosen at the thresholding step. Unfortunately, no data-driven method for determining an optimal threshold has yet been developed, nor has such a threshold been established experimentally. As such, it must be treated as a free parameter. A threshold of 4.5 reveals a single large hyperconnected component and 11 small hypoconnected components in the patient population (Figure 5). These hypoconnected components consist of

1. Left superior frontal gyrus (orbital), left superior frontal gyrus (medial orbital), and left lenticular nucleus (putamen)
2. Left middle frontal gyrus and left caudate nucleus
3. Left Rolandic operculum, left insula, left supramarginal gyrus, left superior temporal gyrus, left middle temporal gyrus, and left temporal pole (middle temporal gyrus)
4. Left middle occipital gyrus, left superior frontal gyrus (medial), left middle occipital gyrus, left inferior occipital gyrus, left and right precuneus, left superior parietal gyrus, and right superior occipital gyrus
5. Left calcarine fissure, left fusiform gyrus, left cuneus, left and right posterior cingulate gyrus, and left superior occipital gyrus
6. Right temporal pole (superior temporal gyrus), right inferior temporal gyrus, and right middle frontal gyrus (orbital)
7. Right middle temporal gyrus and right inferior frontal gyrus (orbital)
8. Right supplementary motor area and right paracentral lobule
9. Right amygdala and right fusiform gyrus
10. Right inferior frontal gyrus (triangular) and right middle frontal gyrus
11. Right inferior frontal gyrus (opercular) and right precentral gyrus

However, it should be emphasized that other settings of the *t*-statistic threshold will produce slightly different results. For example, raising the threshold to *t* = 5 causes the hyperconnected network to fragment into a single large component and two small ones (see Supplementary material). Similarly, one could expect that hypoconnected components would consolidate into fewer, larger networks at lower thresholds.

## Discussion

### Summary

The replacement of *k*-means clustering with ICA and *post hoc* goodness-of-fit metrics with data-driven estimates of dimensionality allowed us to directly quantify and compare whole-brain functional complexity between groups. Independent component analysis has, of course, been used in neuroimaging research for several decades. Thus, one must ask whether LEICA provides an advantage over independent component analysis on the unmodified dynamic functional connectivity array. Of the three low-dimensional spaces evaluated in this study, only the eigenvector-based space captured any differences between patient and control groups. In addition to finding group-level joint entropy alterations, this eigenvector-based space could isolate the dimensions responsible for this increase. Training a model in this space allowed us to capture several connectivity alterations which are well-supported in the neurophysiological literature. This substantial improvement in sensitivity suggests that LEICA will prove a useful tool for future research into functional complexity and dimensionality.

### Phenomenological considerations

Perhaps the greatest advantage of ICA compared to alternative clustering methods, such as *k*-means, is the ability to use metrics which depend on statistical independence. It is probable that this improved resolution will lend itself to other information-theoretic metrics such as functional complexity (Zamora-López et al., 2016) or Granger causality. Such direct comparisons of activity complexity may lead to a deeper understanding of the pathophysiological bases of psychiatric disorders and other neurobehavioral phenomena.

At the phenomenological level, analysis of both leading eigenvectors and unmodified dFC showed higher average entropy in patients than controls. This is in contrast to previous reports of decreased entropy in obsessive-compulsive patients (Aydin et al., 2015), although reports of adolescents with OCD have found increased entropy in networks of cortical and subcortical nodes in the cortico-striatal-thalamo-cortical (CSTC) circuit (Sen et al., 2020). How this apparent increase in complexity—which, in information-theoretic terms, is equivalent to randomness—maps to the well-established tendency of obsessive-compulsive patients to become “stuck” in stereotyped, repetitive patterns will be an interesting topic for future research. It may be that these stereotyped patterns represent a coping mechanism, intended to reduce the randomness of brain activity by imposing control of inputs and responses.

Such a hypothesis receives some support in the fact that the dimension found to increase patient entropy maps to two anticorrelated networks which roughly separate prefrontal-parietal regions vs. subcortical-temporal nodes. Prefrontal and parietal regions exert a top-down inhibitory control on striatal and limbic regions, which has been related to emotion regulation and cognitive control capacities (Ochsner et al., 2012; Etkin et al., 2015). Alterations in such interregional interactions have been associated with mood and anxiety disorders, including OCD (Etkin and Wager, 2007; Picó-Pérez et al., 2017). Decreased order within this network may disrupt top-down inhibition and thus affect emotion regulation and cognitive control, both of which are affected in the context of CSTC dysfunction in OCD (van den Heuvel et al., 2016). Stereotyped, repetitive behaviors—i.e. compulsions—may thus act as a compensatory mechanism by which the brain attempts to impose order on its surroundings.

Interestingly, the affected dimension also contains several occipital nodes. Although the occipital cortex has not typically been considered a core part of neurobiological models of OCD, previous research has shown that such regions and their projections to limbic cortices may play an important role in the induction of increased anxiety levels in patients with contamination obsessions induced by actual or mental images (i.e., intrusive thoughts) of dirt (Göttlich et al., 2014; Moreira et al., 2017). In future research, it may be worth examining whether the patient’s entropic alterations along this dimension correlates with anxiety or compulsive behavior, which could be as measured by e.g. the Hamilton Anxiety Rating Scale (HAM-A) (Hamilton, 1959) or the Yale-Brown Obsessive-Compulsive Scale (Y-BOCS) (Goodman et al., 1989).

### Mechanistic considerations

Regarding the mechanistic analyses, we observe that the generative model recovers a broad network of hyperconnected regions in the patient population. This network includes most nodes in the 90-region AAL atlas and is evident up to a to t-statistic threshold of 4.5. OCD may then be characterized by hyperconnectivity across much of the cortex and subcortical regions. Such a hypothesis would contrast OCD with disorders such as schizophrenia or autism, which appear to be characterized by long-range hypoconnectivity (Friston et al., 2016; Hull et al., 2017); however, previous studies have shown cortical hyperexcitability in patient populations (Cano et al., 2018; Rolls et al., 2020). It is possible that, in keeping with phenomenological results, the hypoconnected components represent regulatory regions whose underperformance encourages hyperactivity. The pallidum, for instance, as part of the CSTC circuitry, is the major inhibitory output from the striatum to the thalamus and the subthalamus, and an increased inhibitory output the from the external globus pallidum to the subthalamus may result in the thalamic and cortical hyperexcitability that has been shown to characterize patients with OCD (Cano et al., 2018). Alterations in the Rolandic operculum, on the other hand, have been related to the impulsive nature of the so-called autogenous obsessions and compulsions in a subgroup of patients with OCD (Subirà et al., 2013), as well as with the premonitory urges associated with tic behaviors and sensory phenomena (Wang et al., 2011) that are observed in a large proportion of patients with OCD (Rosario et al., 2009).

### Limitations and future steps

Three cautionary notes must be added. First, the LEICA method is, by necessity, agnostic as to the true orientation of its communities. Since eigendecomposition and ICA can determine only the orientation of communities relative to each other, not relative to the data itself, LEICA cannot determine which community is “positive” or “negative” in any absolute sense. This may be established by a parallel analysis observing which community is more or less active at any given time; such an analysis is unnecessary for the present purposes.

Second, the Hopf network model should not be considered predictive at the level of individual links. It has been able to replicate known phenomena and mechanisms in past studies (Jobst et al., 2017) and brain-level results, e.g., the widespread cortical hyperconnectivity in obsessive-compulsive patients, appear robust. However, the Hopf oscillator remains an idealized simplification of neural dynamics. To predict neurobiological mechanisms would require both more detailed data and a more sophisticated model, e.g., a model incorporating transmission delays and neuromodulation. Link-level model results in this paper should thus be considered starting points for future research rather than forming hard conclusions themselves.

Finally, while the network-based statistic (NBS) is a well-established method, its results remain dependent on the choice of *t*-statistic threshold employed. This does not affect the power of the results, only the effect size of the results reported. Unfortunately, no data-driven method has yet been established for determining an appropriate threshold. However, studying which connections survive the different thresholds allows us to partially quantify the group-level effect size.

In addition to these general concerns, the present model fits the control group considerably better than the patient group. It is not immediately clear to the authors why this is the case, as both groups undergo identical procedures. That the model returns meaningful results despite this poor performance suggests that improving the fitting procedure’s performance may yield entirely novel insights. The question of model optimization will be of major interest in future studies.

The widespread alterations in cortical connectivity likely affect activity propagation and organization. While such alterations were outside the scope of this study, they are of great interest to the understanding of OCD’s functionality. Leveraging established network analyses frameworks, such as community detection or node centrality measures, may provide further insights into the cortical activity adaptations of OCD, and potentially in related disorders such as anxiety and depression.

## Conclusion

The search for a natural low-dimensional space for the analysis of functional connectivity dynamics remains an active area of research. We present a novel method based on established theory to map functional activity to such a space. The resulting space ensures interdimensional statistical independence, which allows the quantification and direct comparison of information content (randomness) between groups and subjects. Comparisons with classic independent component analysis shows that LEICA preserves functional complexity while increasing sensitivity and power. This increased power allows LEICA to recover evidence supporting several extant hypotheses on the causes of obsessive-compulsive disorder, most notably the importance of top-down control as exerted by prefrontal and parietal regions on the limbic system. Training a generative model in this space similarly recovers known functional characteristics of OCD, e.g., broad cortical hyperconnectivity, and highlights specific connections as targets for future studies. Given these results and its novel ability to directly compare information content, we anticipate that the LEICA framework and its extensions will become a crucial tool in the ongoing efforts to quantify and explain the connectivity substates of the brain in both human and nonhuman studies.

## Data availability statement

The raw data supporting the conclusions of this article will be made available by the authors, without undue reservation. The code used in this study is available online at https://github.com/decolab/LEICA, https://github.com/decolab/Functions and https://github.com/decolab/Effective-Connectivity--Hopf.

## Ethics statement

The studies involving human participants were reviewed and approved by Ethics Committee of the Hospital de Braga, Portugal. The patients/participants provided their written informed consent to participate in this study.

## Author contributions

DB the primary author of this article developed and executed the analysis in, wrote the text of, and generated all figures in this report. CS-M provided neurobiological and physiological analysis for this report. JC provided the code and explanation for the LEiDA algorithm, which the primary author developed into the methods described in this article. PMore and PMorg collected the data analyzed in this report, as detailed in the methods section. GD, proposed this study to the primary author and supervised the course of this research. All authors contributed to the article and approved the submitted version.

## Supplementary material

Supplementary Figure 1 To compute time-resolved functional connectivity (dynamic functional connectivity, or dFC), each bandpass-filtered regional time series is converted into an analytic signal using the Hilbert transform. Euler’s formula converts this analytic signal into a time-resolved phase signal **(A)**. For each time point, the phase signals of all regions are sampled **(B)** and the cosine distance between each pair of regions is computed to produce an instantaneous functional connectivity matrix **(C)**. The leading eigenvector **V_1_** of this functional connectivity matrix is then isolated **(D)**. Repeating this process for all time points and subjects across the dataset results in a 2-D array **E** of leading eigenvectors **(E)**. Running an eigendecomposition on **E**’s autocorrelation matrix and counting the number of eigenvalues greater than the upper bound of the Marčenko-Pastur distribution reveals the number of dimensions necessary to describe the nonrandom activity in **E**. This figure displays all regions with a z-score higher than z = 1 in full color.

Supplementary Figure 2 Results from the network-based statistic. A t-statistic threshold of 4.0 returns six connected components **(C)**, visualized together as a connectivity matrix **(A)** and in cortical space **(B)**. Cyan links indicate that the connection is stronger in OCD patients than in healthy controls, while magenta links indicate the converse. Although only one connected component displays increased strength in patients, this component includes all 90 of the cortical nodes in the AAL parcellation, suggesting that obsessive-compulsive disorder may be characterized by widespread cortical hyperconnectivity. Control-biased components, by contrast, consist of between three to nine links, with larger components tending to concentrate in small topographical areas. Notably, many regions displaying depressed connectivity in patients are known to be involved in top-down control and impulse inhibition. OCD may thus be characterized by localized disruptions in top-down inhibitory activity, which may explain the widespread hyperconnectivity observed in patients.

Supplementary Figure 3 Results from the network-based statistic. A t-statistic threshold of 5.0 returns 11 connected components **(C)**, visualized together as a connectivity matrix **(A)** and in cortical space **(B)**. Cyan links indicate that the connection is stronger in OCD patients than in healthy controls, while magenta links indicate the converse. At this threshold, the connected component displaying increased strength in patients breaks into three sections, with the largest containing 78 of the 90 nodes in the AAL parcellation. This is considerably larger than the other two control-biased components, which have one and two links respectively. The eight surviving control-biased components consist of up to three links.

Supplementary Figure 4 Results from the network-based statistic. A t-statistic threshold of 5.5 returns 16 connected components **(C)**, visualized together as a connectivity matrix **(A)** and in cortical space **(B)**. Cyan links indicate that the connection is stronger in OCD patients than in healthy controls, while magenta links indicate the converse. At this threshold, the connected component displaying increased strength in patients breaks into 10 sections, with the largest containing 33 of the 90 regions of the AAL. The six control-biased components consist of up to two links. The fall in number of control-based components with increasing t-threshold further indicates that OCD is characterized by excessive connectivity across the cortex.

Supplementary Figure 5 Results from the network-based statistic. A t-statistic threshold of 6.0 returns 17 connected components **(C)**, visualized together as a connectivity matrix **(A)** and in cortical space **(B)**. Cyan links indicate that the connection is stronger in OCD patients than in healthy controls, while magenta links indicate the converse. At this threshold, the connected component displaying increased strength in patients breaks into 14 sections. Most of these consist of only one or two links. The two largest consist of a 14-region and nine-region chain, respectively. Only three control-biased components survive, neither of which exceeds a single link in size. It thus appears that OCD is characterized by excessive connectivity in patients.
