# Corrigendum: Complexity changes in functional state dynamics suggest focal connectivity reductions

**DOI:** 10.3389/fnhum.2023.1275387

**Published:** 2023-10-11

**Authors:** David Sutherland Blair, Carles Soriano-Mas, Joana Cabral, Pedro Moreira, Pedro Morgado, Gustavo Deco

**Affiliations:** ^1^Facultad de Comunicación, Universitat Pompeu Fabra, Barcelona, Spain; ^2^Psychiatry and Mental Health Group, Neuroscience Program, Institut d'Investigació Biomèdica de Bellvitge, Barcelona, Spain; ^3^Network Center for Biomedical Research on Mental Health, Carlos III Health Institute, Madrid, Spain; ^4^Department of Social Psychology and Quantitative Psychology, Universitat de Barcelona, Barcelona, Spain; ^5^Life and Health Sciences Research Institute, School of Medicine, University of Minho, Braga, Portugal; ^6^ICVS/3B's, PT Government Associate Laboratory, Braga, Portugal; ^7^Psychological Neuroscience Lab, CIPsi, School of Psychology, University of Minho, Braga, Portugal; ^8^Clinical Academic Center—Braga, Braga, Portugal; ^9^Institució Catalana de Recerca i Estudis Avançats, Barcelona, Spain; ^10^Department of Neuropsychology, Max Planck Institute for Human Cognitive and Brain Sciences, Leipzig, Germany; ^11^School of Psychological Sciences, Monash University, Clayton, VIC, Australia

**Keywords:** LEiDA, Hopf bifurcation, whole-brain model, obsessive-compulsive disorder, independent component analysis, eigendecomposition, Shannon entropy, network-based statistic

In the published article, there was an error in the Funding statement (a project reference is missing from the funding statement):

G. D. and D.B. were supported by the Spanish national research project (ref. PID2019-105772GB-I00AEI/10.13039/501100011033) funded by the Spanish Ministry of Science, Innovation and Universities (MCIU), State Research Agency (AEI).

The correct Funding statement appears below.

This work was funded by ICVS Scientific Microscopy Platform, member of the National infrastructure PPBI–Portuguese Platform of Bioimaging (PPBI-POCI01 0145-FEDER-022122); by National funds, through the Foundation for Science and Technology (FCT)–project UIDB/50026/2020 and UIDP/50026/2020; and by the project NORTE-01-0145-FEDER-000039, supported by Norte Portugal Regional Operational Programme (NORTE 2020), under the PORTUGAL 2020 Partnership Agreement, through the European Regional Development Fund (ERDF). GD and DB were supported by the Spanish national research project (ref. PID2019-105772GB-I00/AEI/10.13039/501100011033) funded by the Spanish Ministry of Science, Innovation and Universities (MCIU) and State Research Agency (AEI).

The authors apologize for this error and state that this does not change the scientific conclusions of the article in any way. The original article has been updated.

