# Supplementary material for: Complexity changes in functional state dynamics suggest focal connectivity reductions
Source: Front Hum Neurosci. 2022 Sep 23;16:958706. doi: 10.3389/fnhum.2022.958706 (PMC9540393; doi:10.3389/fnhum.2022.958706)
Supplement: Supplementary Figure 1 — To compute time-resolved functional connectivity (dynamic functional connectivity, or dFC), each bandpass-filtered regional time series is converted into an analytic signal using the Hilbert transform. Euler’s formula converts this analytic signal into a time-resolved phase signal (A). For each time point, the phase signals of all regions are sampled (B) and the cosine distance between each pair of regions is computed to produce an instantaneous functional connectivity matrix (C). The leading eigenvector V1 of this functional connectivity matrix is then isolated (D). Repeating this process for all time points and subjects across the dataset results in a 2-D array E of leading eigenvectors (E). Running an eigendecomposition on E’s autocorrelation matrix and counting the number of eigenvalues greater than the upper bound of the Marčenko-Pastur distribution reveals the number of dimensions necessary to describe the nonrandom activity in E. This figure displays all regions with a z-score higher than z = 1 in full color. [file Data_Sheet_1.PDF]

Supplementary Material

1 Supplementary Data

2 Supplementary Figures and Tables

2.1 Supplementary Figures

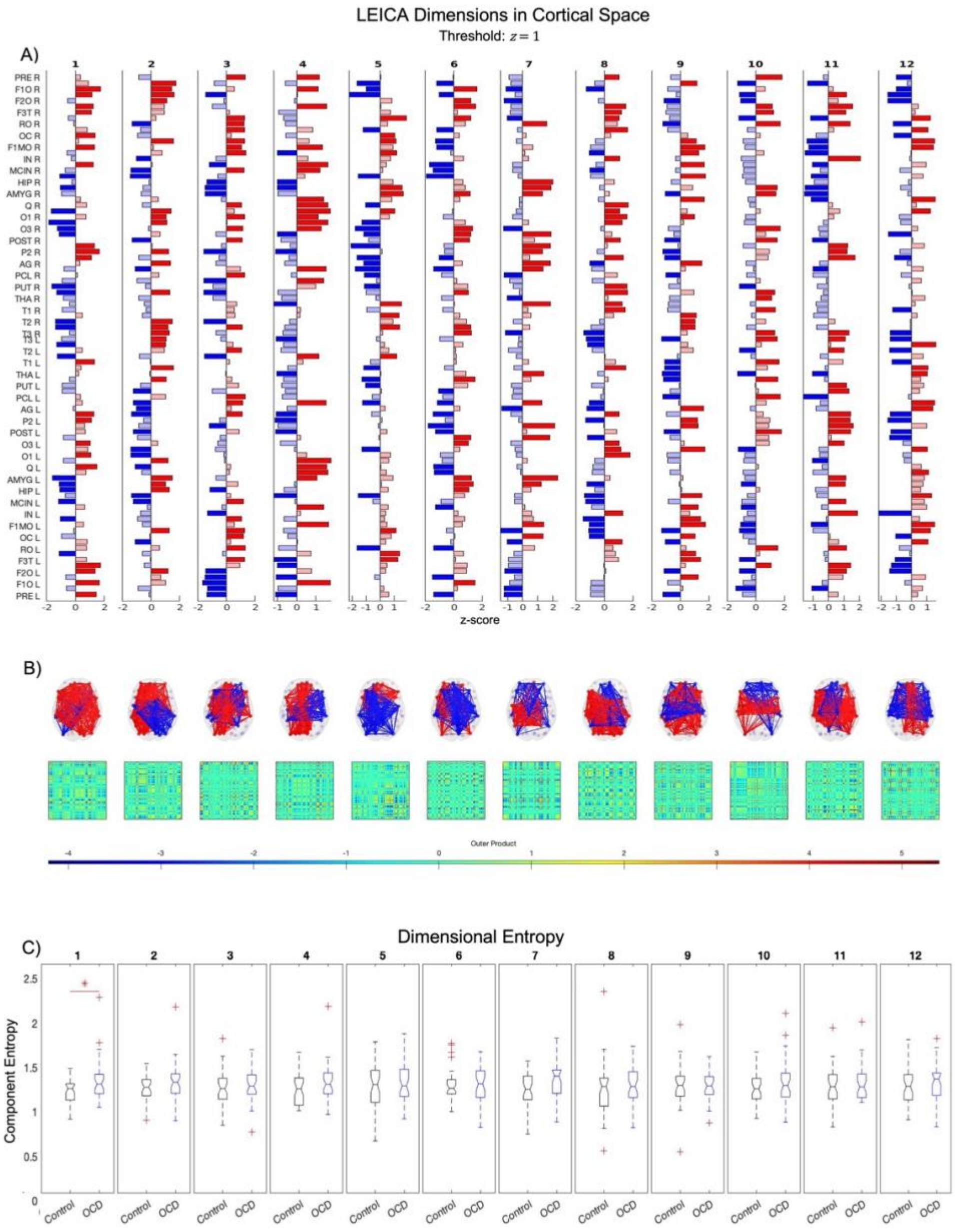

Supplementary Figure 1.

To compute time-resolved functional connectivity (dynamic functional connectivity, or dFC), each bandpass-filtered regional time series is converted into an analytic signal using the Hilbert transform. Euler's formula converts this analytic signal into a time-resolved phase signal (**A**). For each time point, the phase signals of all regions are sampled (**B**) and the cosine distance between each pair of regions is computed to produce an instantaneous functional connectivity matrix (**C**). The leading eigenvector $\mathbf{V_1}$ of this functional connectivity matrix is then isolated (**D**). Repeating this process for all time points and subjects across the dataset results in a 2-D array $\mathbf{E}$ of leading eigenvectors (**E**). Running an eigendecomposition on $\mathbf{E}$'s autocorrelation matrix and counting the number of eigenvalues greater than the upper bound of the Marčenko-Pastur distribution reveals the number of dimensions necessary to describe the nonrandom activity in $\mathbf{E}$. This figure displays all regions with a z-score higher than $z = 1$ in full color.

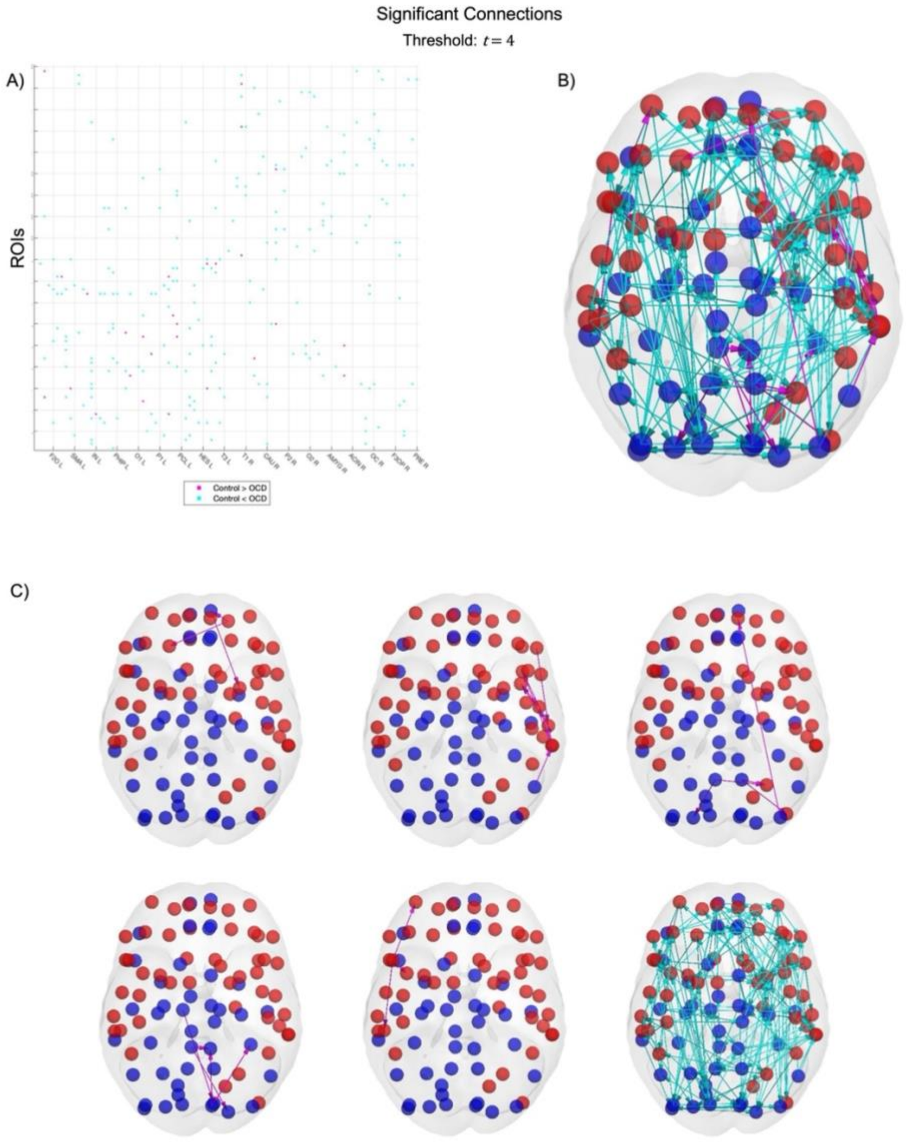

Supplementary Figure 2.

Results from the network-based statistic. A t-statistic threshold of 4.0 returns six connected components (**C**), visualized together as a connectivity matrix (**A**) and in cortical space (**B**). Cyan links indicate that the connection is stronger in OCD patients than in healthy controls, while magenta links indicate the converse. Although only one connected component displays increased strength in patients, this component includes all 90 of the cortical nodes in the AAL parcellation, suggesting that obsessive-compulsive disorder may be characterized by widespread cortical hyperconnectivity. Control-biased components, by contrast, consist of between three to nine links, with larger components tending to concentrate in small topographical areas. Notably, many regions displaying depressed connectivity in patients are known to be involved in top-down control and impulse inhibition. OCD may thus be characterized by localized disruptions in top-down inhibitory activity, which may explain the widespread hyperconnectivity observed in patients.

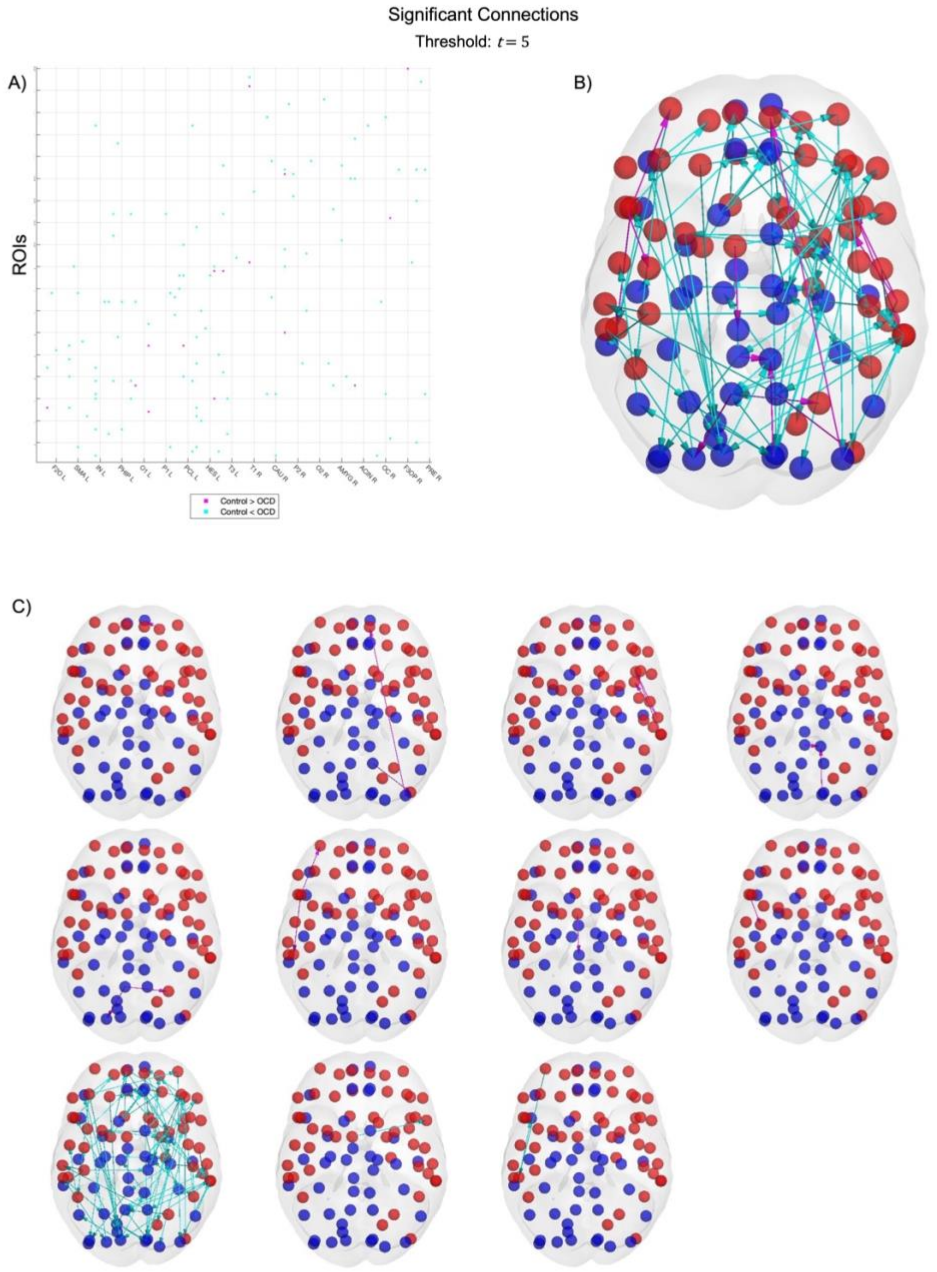

Supplementary Figure 3.

Results from the network-based statistic. A t-statistic threshold of 5.0 returns eleven connected components (**C**), visualized together as a connectivity matrix (**A**) and in cortical space (**B**). Cyan links indicate that the connection is stronger in OCD patients than in healthy controls, while magenta links indicate the converse. At this threshold, the connected component displaying increased strength in patients breaks into three sections, with the largest containing 78 of the 90 nodes in the AAL parcellation. This is considerably larger than the other two control-biased components, which have one and two links respectively. The eight control-biased components consist of up to three links.

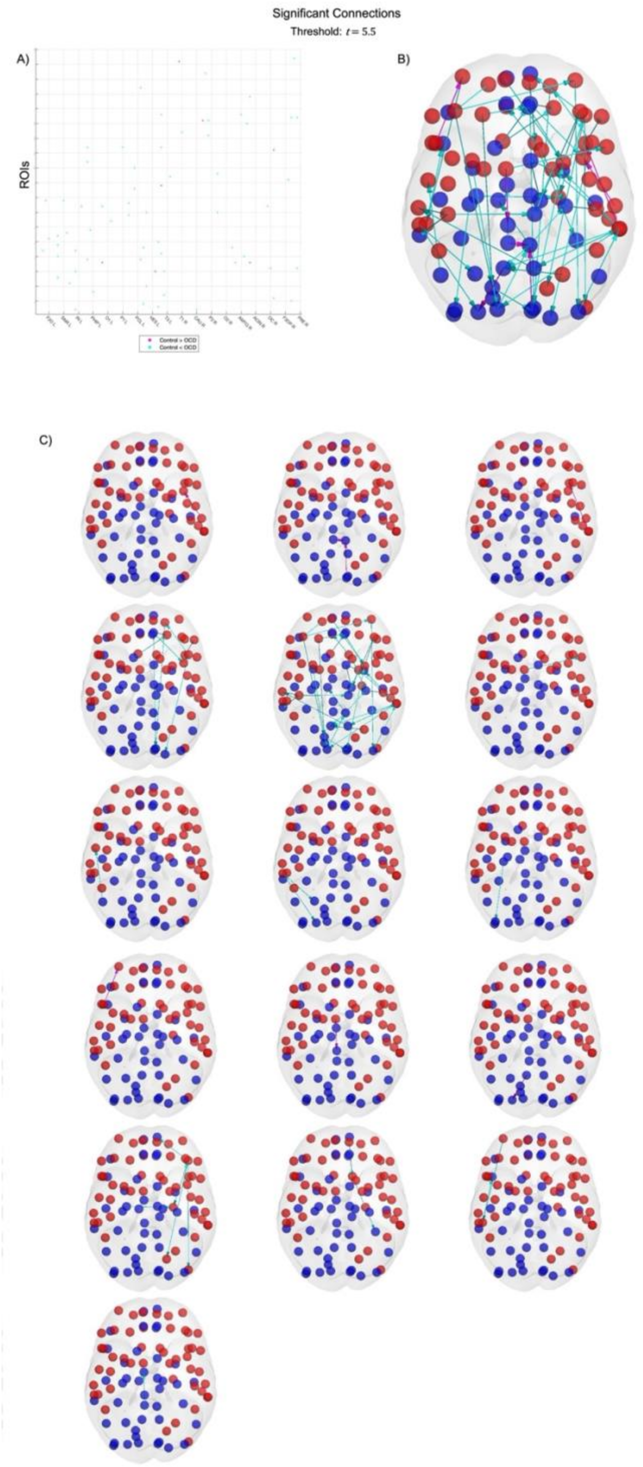

Supplementary Figure 4

Results from the network-based statistic. A t-statistic threshold of 5.5 returns sixteen connected components (**C**), visualized together as a connectivity matrix (**A**) and in cortical space (**B**). Cyan links indicate that the connection is stronger in OCD patients than in healthy controls, while magenta links indicate the converse. At this threshold, the connected component displaying increased strength in patients breaks into ten sections, with the largest containing 33 of the 90 regions of the AAL. The six control-biased components consist of up to two links. The fall in number of control-based components with increasing t-threshold further indicates that OCD is characterized by excessive connectivity across the cortex.

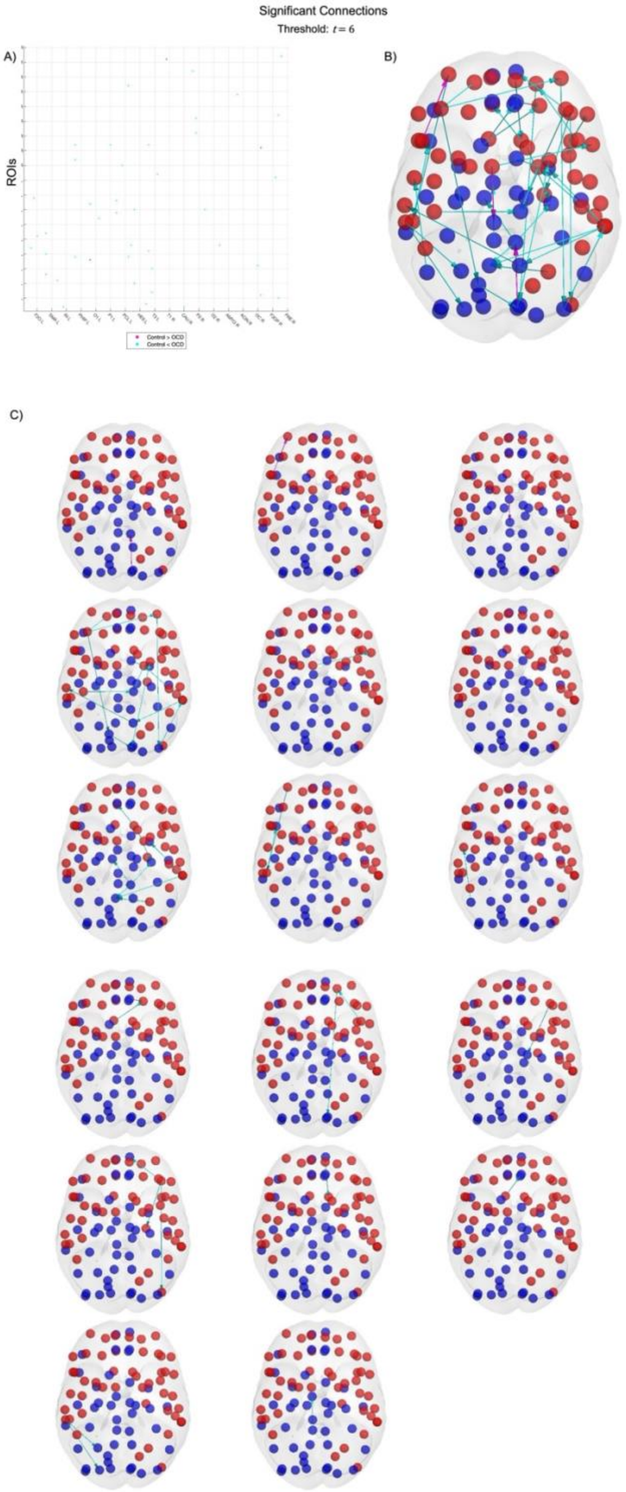

Supplementary Figure 5

Results from the network-based statistic. A t-statistic threshold of 6.0 returns seventeen connected components (**C**), visualized together as a connectivity matrix (**A**) and in cortical space (**B**). Cyan links indicate that the connection is stronger in OCD patients than in healthy controls, while magenta links indicate the converse. At this threshold, the connected component displaying increased strength in patients breaks into fourteen sections. Most of these consist of only one or two links. The two largest consist of a fourteen-region and nine-region chain, respectively. Only three control-biased components survive, neither of which exceeds a single link in size. It thus appears that OCD is characterized by excessive connectivity in patients.
